# Immunohistochemical evaluation of molecular subtypes in muscle invasive bladder cancer with demographic correlation

**DOI:** 10.1186/s12885-026-16476-3

**Published:** 2026-07-10

**Authors:** Pranita Mohanty, Megha Subhangini

**Affiliations:** Dept of Pathology, IMS & SUM Hospital, Siksha’O’Anusandhan University, Bhubaneswar, Odisha 751003 India

**Keywords:** Bladder cancer, Muscle-invasive bladder cancer, Immunohistochemistry, Molecular subtypes, GATA3, CK5/6, FGFR3, HER2

## Abstract

**Background:**

Muscle-invasive bladder cancer (MIBC) is a biologically heterogeneous malignancy with variable clinical outcomes. Molecular subtyping has improved understanding of tumor biology and therapeutic stratification.

**Materials and methods:**

This retrospective study included 64 cases of MIBC. Immunohistochemistry (IHC) was performed using GATA3, CK5/6, p53, and FGFR3 to classify tumours into molecular subtypes. HER2 expression was also evaluated and correlated with clinicopathological parameters.

**Results:**

Luminal subtype was predominant (76.6%). CK5/6 positivity was observed in 38.3% of cases. Aberrant p53 expression was noted in 79.8% of tumours. FGFR3 expression was detected in 17% of cases and was significantly associated with luminal subtype. HER2 overexpression was more frequent in luminal tumours. Basal tumours showed better response to neoadjuvant chemotherapy, whereas luminal tumours demonstrated higher FGFR3 and HER2 expression. p53-altered tumours showed features of chemoresistance.

**Conclusion:**

IHC-based molecular classification is a practical surrogate for transcriptomic profiling in MIBC and may assist in prognostication and therapeutic decision-making.

## Introduction

Urothelial carcinoma (UC) is the most common histological subtype of bladder cancer, accounting for approximately 80–90% of all cases worldwide [1–3]. Despite advances in diagnosis and treatment, bladder cancer remains a major cause of cancer-related morbidity and mortality due to its marked biological and clinical heterogeneity [2–4]. Traditionally, UC is classified into non-muscle-invasive bladder cancer (NMIBC) and muscle-invasive bladder cancer (MIBC), with the latter associated with poorer prognosis and higher risk of progression and metastasis [2–4].

Recent advances in molecular oncology have demonstrated that MIBC is not a single disease entity but comprises distinct molecular subtypes with differing biological behaviour, prognosis, and therapeutic response [1, 5–8]. Among these, luminal and basal subtypes are the most clinically relevant and reflect gene expression patterns similar to the normal urothelial differentiation [1, 5, 6, 9]. Additional subgroups such as the p53-like subtype have also been described and are associated with chemoresistance and poor response to neoadjuvant chemotherapy [7, 10, 11].

Although gene expression profiling remains the gold standard for molecular classification, its routine clinical application is limited due to cost and technical complexity [6–9]. Therefore, immunohistochemistry (IHC) has emerged as a practical surrogate method for molecular subtyping [12, 13]. A limited panel including GATA3 and FGFR3 (luminal markers), CK5/6 (basal marker), and p53 (p53-like phenotype) has demonstrated strong concordance with transcriptomic classification [12, 13].

In addition to classification, these biomarkers have prognostic and therapeutic significance. FGFR3 alterations are enriched in luminal tumors and represent actionable targets in bladder cancer [14, 15]. HER2 overexpression has also been identified in a subset of urothelial carcinoma and represents a potential therapeutic target [16–20]. Thus, integrated molecular and immunohistochemical profiling may improve patient stratification and support personalized therapy [21, 22].

The present study aimed to classify MIBC using IHC markers (GATA3, CK5/6, p53, FGFR3) and evaluate their clinicopathological correlation, along with HER2 expression across molecular subtypes [18–22].

## Materials and methods

### Study design and patient selection

This retrospective observational study was conducted in the Department of Pathology at a tertiary care teaching hospital over a three-year period. A total of 64 patients with histopathologically confirmed MIBC were included.

Patients with non-muscle-invasive disease (Ta, T1, carcinoma in situ), UC with metastasis(T3,T4&locoregional lymph nodal spread cases) at presentation, or inadequate tissue samples were excluded.

### Histopathological evaluation

All cases were reviewed on haematoxylin and eosin-stained sections. Tumors were graded according to WHO classification and staged using the TNM system. Features such as lymphovascular invasion and perineural invasion were recorded.

### Immunohistochemistry

IHC was performed using formalin-fixed paraffin-embedded tissue sections on a Ventana Benchmark XT system. Primary antibodies included GATA3, CK5/6, p53, FGFR3, and HER2.

Interpretation criteria were standardized as follows:

- GATA3: Strong nuclear staining in ≥ 50% tumor cells indicates positivity
- CK5/6: Cytoplasmic staining in > 20% tumor cells indicates basal phenotype
- p53: Aberrant(mutant) pattern defined as either diffuse strong nuclear positivity or complete absence
- FGFR3: Cytoplasmic/membranous positivity indicates expression
- HER2: Scored 0–3 + according to ASCO/CAP guidelinesthat of Breast.

### Molecular subtyping

Tumors were classified as:

- Luminal: GATA3 positive, CK5/6 negative
- Basal: CK5/6 positive, GATA3 negative
- p53-like: Aberrant p53 expression pattern
- Null type: Negative for both GATA3 and CK5/6

### Statistical analysis

The results were analyzed using descriptive statistics and comparisons among various groups. Categorical data were summarized as proportions and percentages (%) and quantitative data were summarized as mean ± SD. It’s a hypothesis test used to assess the relationship between two categorical variables. A two-sided (*p* < 0.05) was considered statistically significant. The analysis was done in IBM SPSS version 23.

Ethical approval was obtained by institutional Ethics committee.

INSTITUTE OF MEDICAL SCIENCES (IMS) AND SUM HOSPITAL.

Siksha ‘O’ Anusandhan (Deemed to be University).

(Accredited by NACC of UGC with ‘A’ grade)

IEC/IMS &SH (SOA) with regd no.- ECR/627/Inst/OR/2014/RR – 20.

- All the methods were carried out in accordance with relevant guidelines and regulations. All experimental protocols were approved by the said committee.
- Prior informed consent was taken from all participants/ or their legal guardian(s). Human Ethics were not violated as no new experimental procedure done & this work was a part of their usual procedure for treatment purpose.

## Results

The study included 64 cases of muscle-invasive bladder carcinoma (MIBC). The most common age group was 60–70 years (40.6%), with a marked male predominance (90.6%). The most common clinical presentation was painless haematuria, observed in 46 cases (71.9%), followed by intermittent haematuria in 12.5% of cases.

**Fig. 1:**
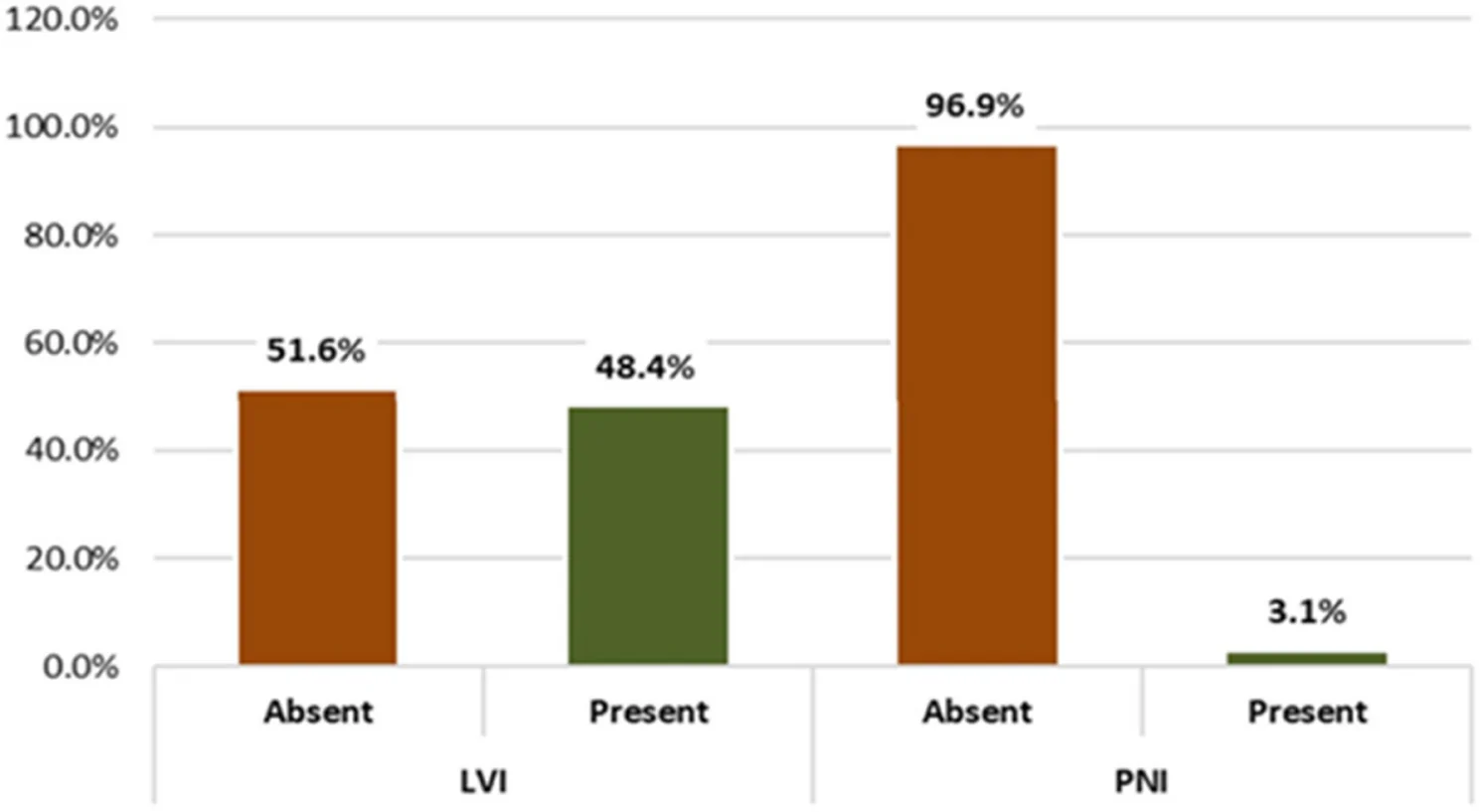
Distribution of LVI and PNI

The most frequent tumor location was the anterior bladder wall (32.8%), followed by the lateral wall (25.5%). Histopathological evaluation revealed ten cases of papillary urothelial carcinoma with squamous differentiation and one case of urothelial carcinoma with plasmacytoid differentiation, rest all were of classical invasive urothelial carcinomas. Lymphovascular invasion (LVI) was seen in 33 cases (48.4%), whereas perineural invasion (PNI) was identified in 2 cases (3.1%), indicating a predominantly aggressive tumor profile (Table 1; Fig. 1).

**Table 1:** Clinicopathological characteristics of cases

Characteristics	No. of patients(*n* = 64)	Percentage(%)
Age		
< 60	21	32.8
≥ 60	43	67.1
Sex		
Male	58	90.6
Female	06	9.4
Specimen type		
TURBT	52	81.25
Radical cystoprostatectomy	12	18.75
Radiological tumor location		
Anterior UB wall	21	32.8
Lateral UB wall	16	25.0
LVI		
Present	31	48.4
Absent	33	51.6
PNI		
Present	02	3.1
Absent	62	96.9

**Fig. 2:**
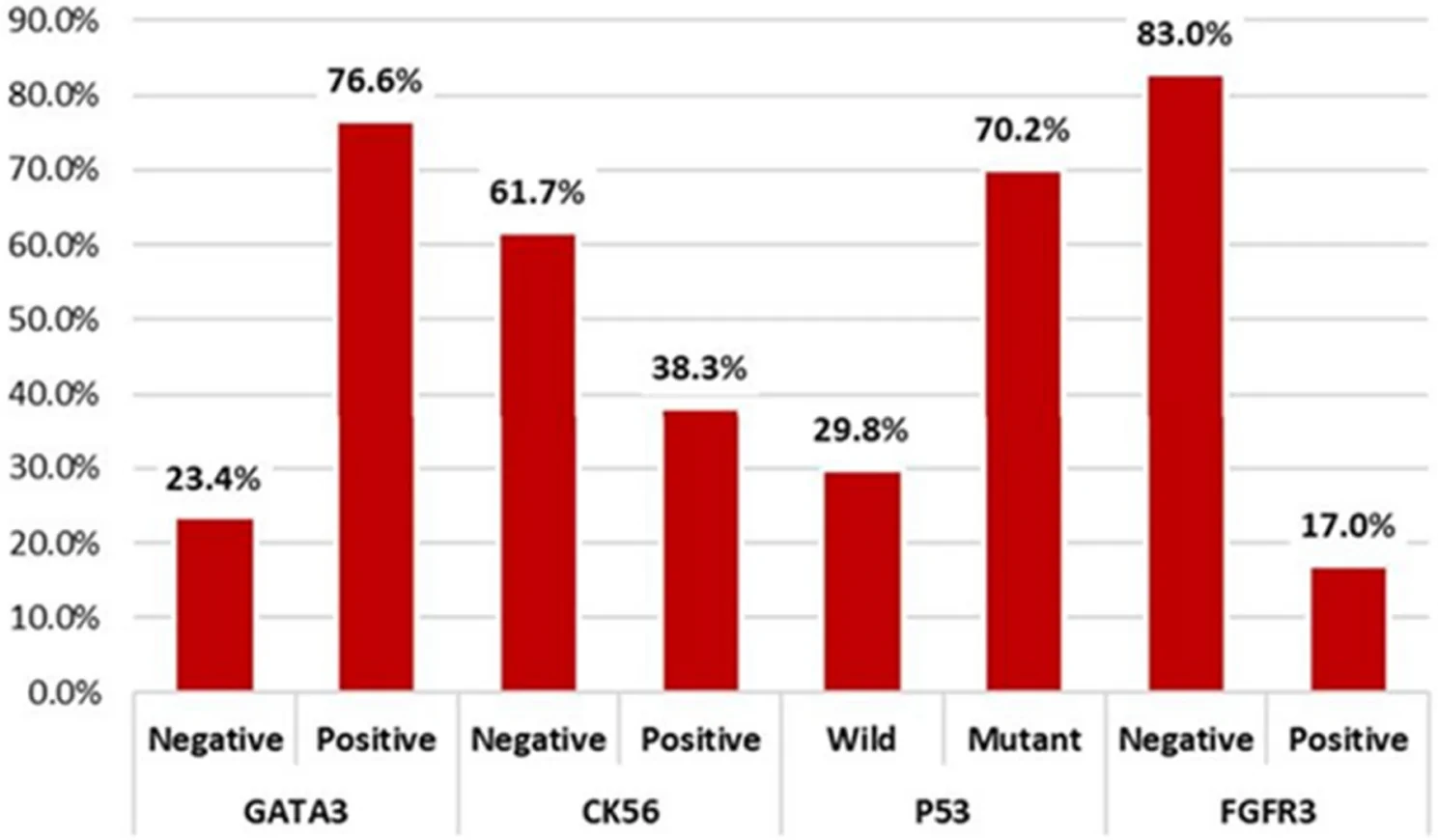
Distribution of Immunohistochemicalstains

**Fig. 3:**
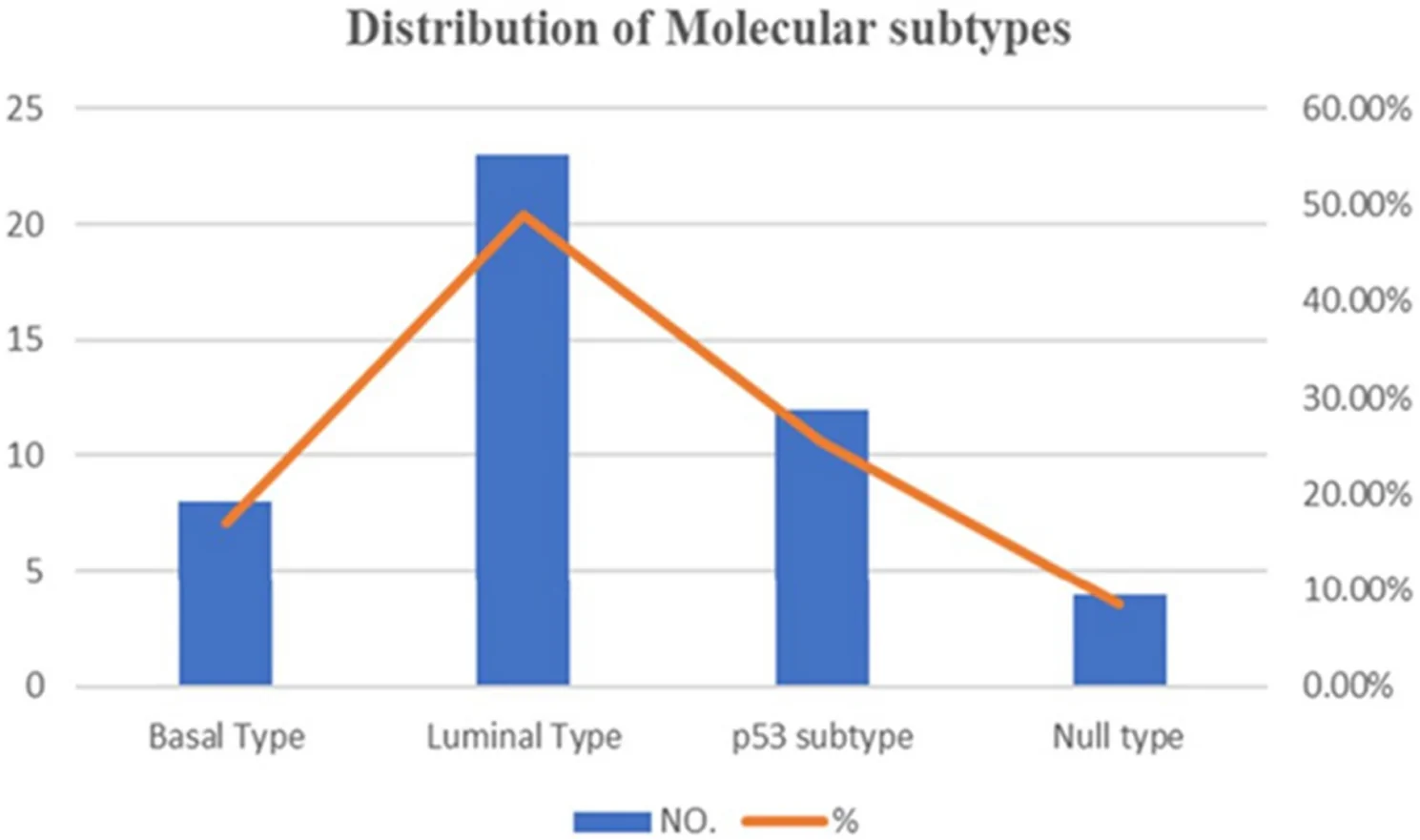
Molecular subtypes

**Fig. 4:**
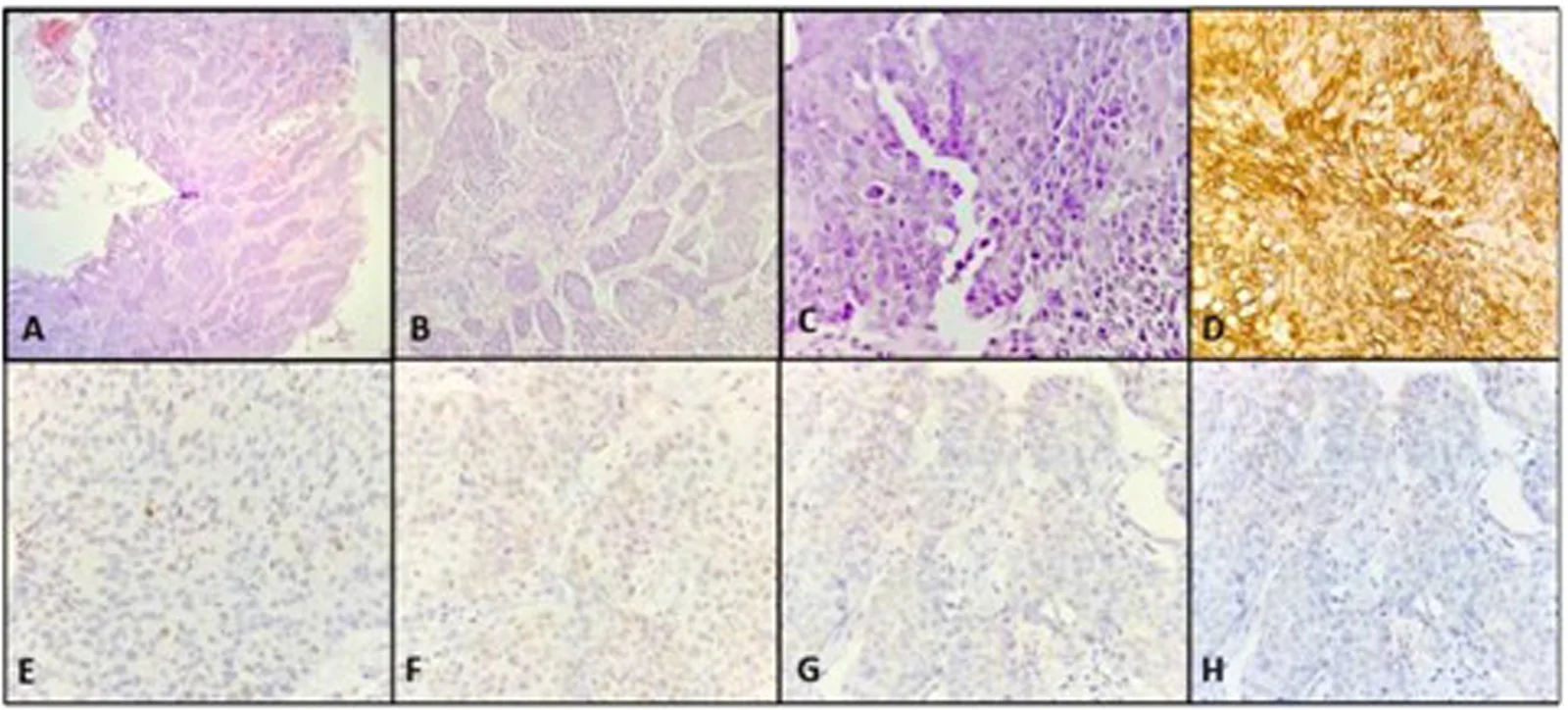
**A**-**C** Photomicrograph showing High grade invasive papillary urothelial carcinoma with squamous differentiation. [Fig.1A-4x,1B-10x,1C-40x, H&E], (**D**-**H**) (40x) Photomicrograph showing IHC for CK5/6 positive, p53 mutant, GATA3, FGFR3 & HER2 negative (score 0) respectively

**Fig. 5:**
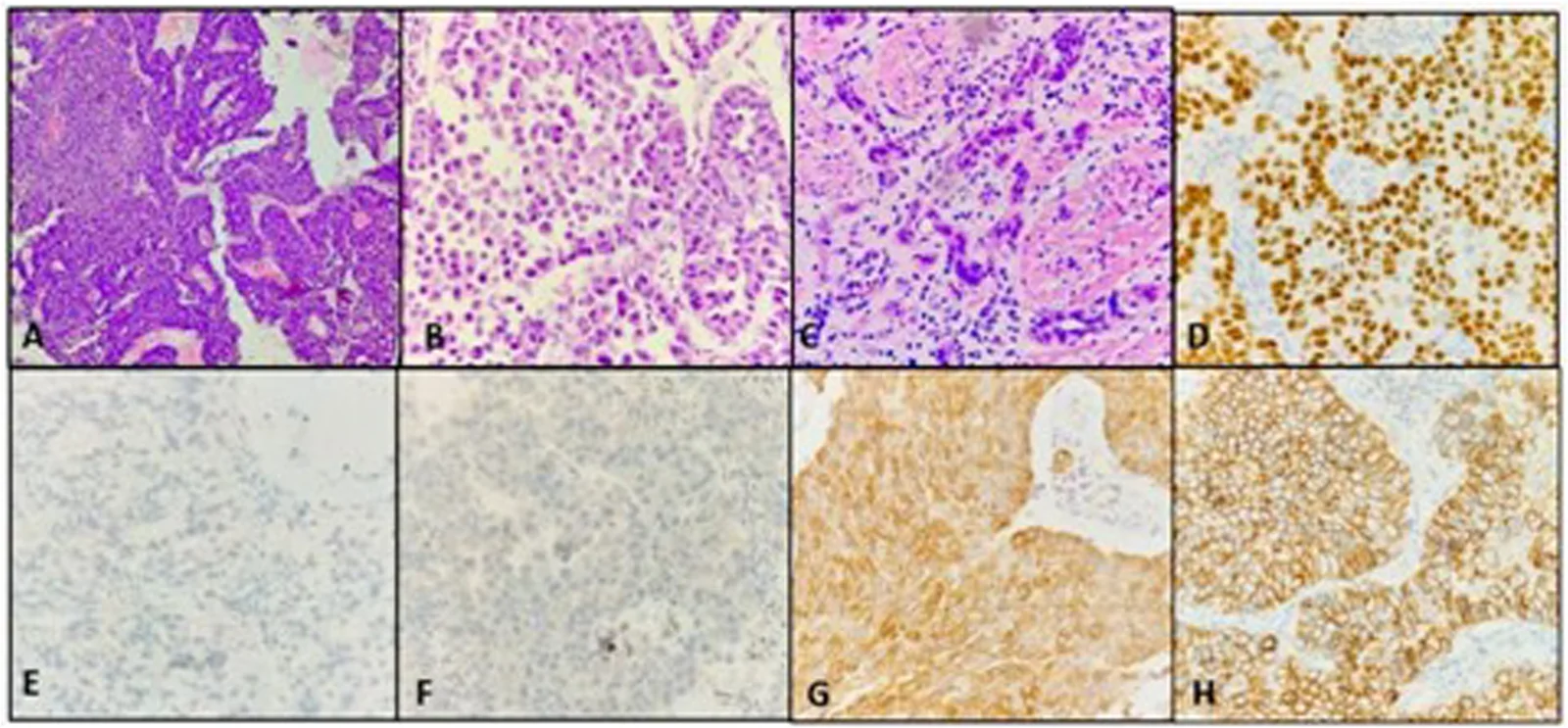
**A**-**D** Photomicrograph showing High grade invasive papillary urothelial carcinoma and deep muscle invasion. [2A-4x, 2B-10x, 2C -40x (H&E)]. **E**-**H** Photomicrograph of IHC showing GATA3 positive, CK5/6 negative, p53 wild type, FGFR3 positive and HER2 positive (score 3+) (2E-I-40x)

**Fig. 6:**
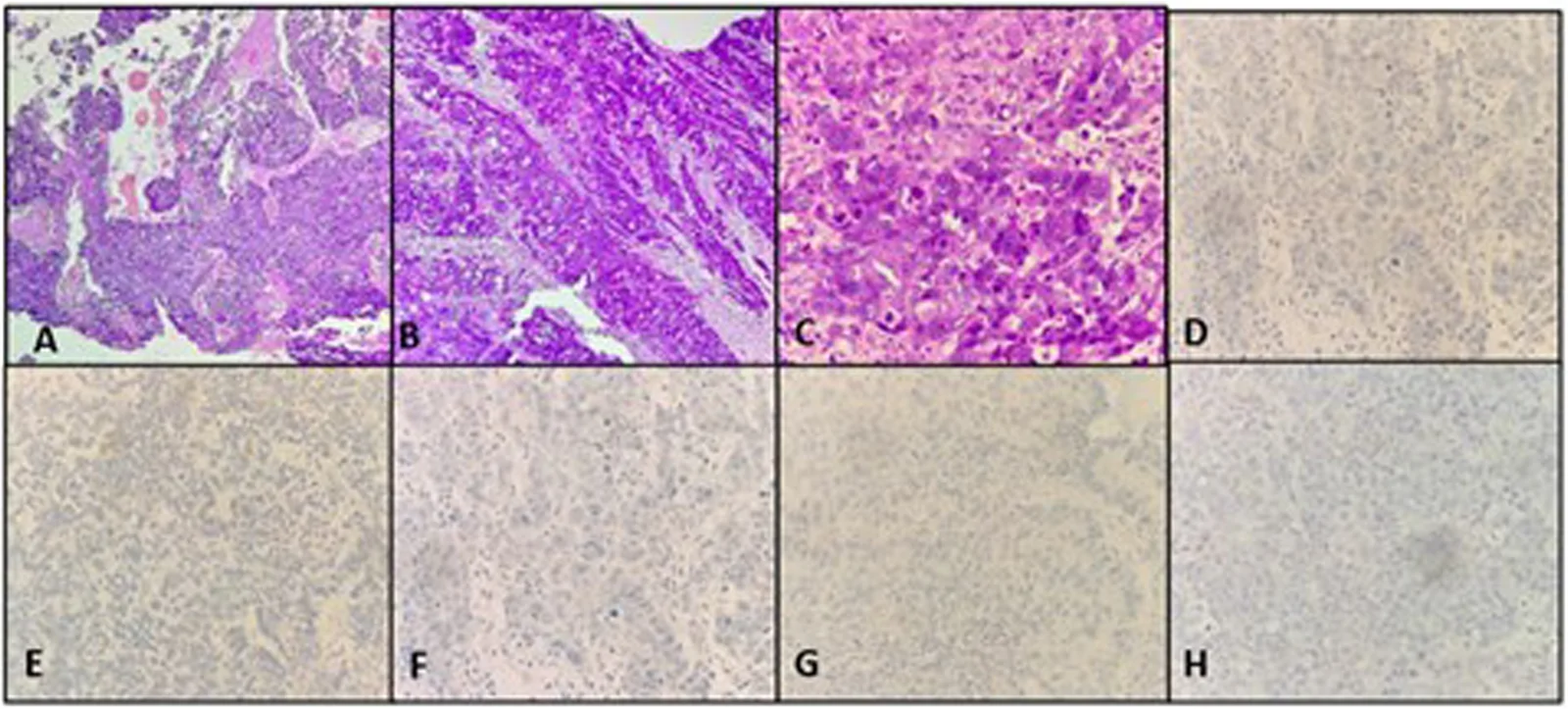
**A**-**D** Photomicrograph showing invasive papillary urothelial carcinoma, high grade [3A- 4x, 3B-10x, 3C – 40x (H&E)]. **E**-**H** Photomicrograph of IHC showing GATA3, CK5/6 negative, p53 mutant, FGFR3 and HER2 negative (score 0) (40x) respectively

**Fig. 7:**
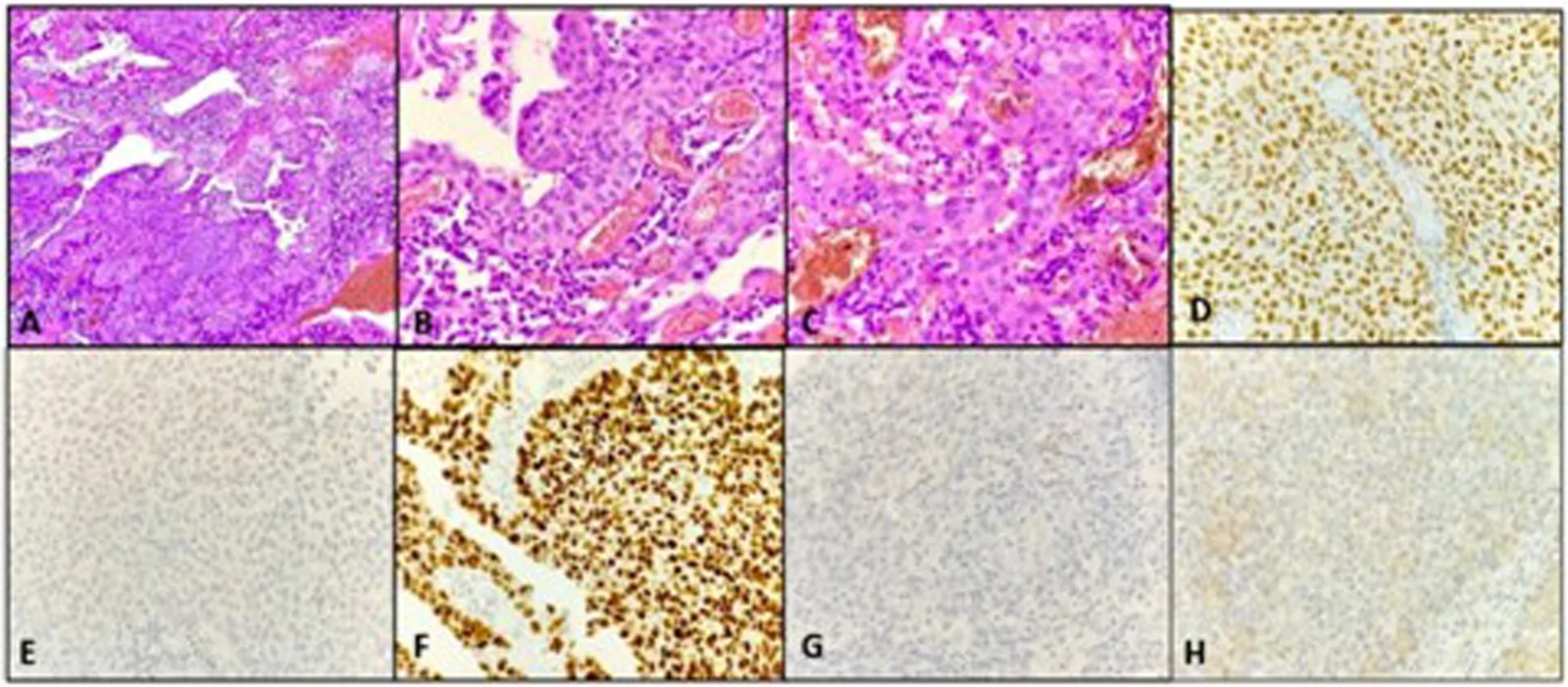
**A**-**D** Photomicrograph showing papillary urothelial carcinoma Tumor cells exhibit moderate to marked nuclear pleomorphism with few mitotic figures and many congested blood vessels [4A-4X,4B & C-40X (H&E)]. Photomicrograph of IHC (E-H) showing GATA3 positive, CK5/6 negative, p53 mutant, FGFR3 negative and HER2 negative (score 0) (40x) respectively

Based on immunohistochemical expression patterns, cases were categorized into luminal, basal, p53-altered, and null subtypes (Tables 2 and 3; Figs. 2 and 3). GATA3 expression was observed in 76.6% of cases, predominantly corresponding to the luminal subtype. CK5/6 positivity was detected in 38.3% of cases, consistent with basal differentiation. Aberrant p53 expression (mutant pattern) was observed in 79.8% of cases, while a wild-type staining pattern (scattered weak nuclear positivity) was seen in 29.8% of cases (Figs. 4, 5, 6 and 7).

**Table 2:** Distribution of immunohistochemicalstains

Immunohistochemicalstains		No.	%
GATA3	Negative	11	23.4%
	Positive	36	76.6%
CK56	Negative	29	61.7%
	Positive	18	38.3%
P53			
	Wild	14	29.8%
	Mutant	33	70.2%
FGFR3	Negative	39	83.0%
	Positive	8	17.0%

**Table 3:** Molecular subtypes

MOLECULARTYPE	No.	%
Nulltype	4	8.5%
BasalType	8	17.0%
LuminalType	23	48.9%
p53subtype	12	25.5%

**Fig. 8:**
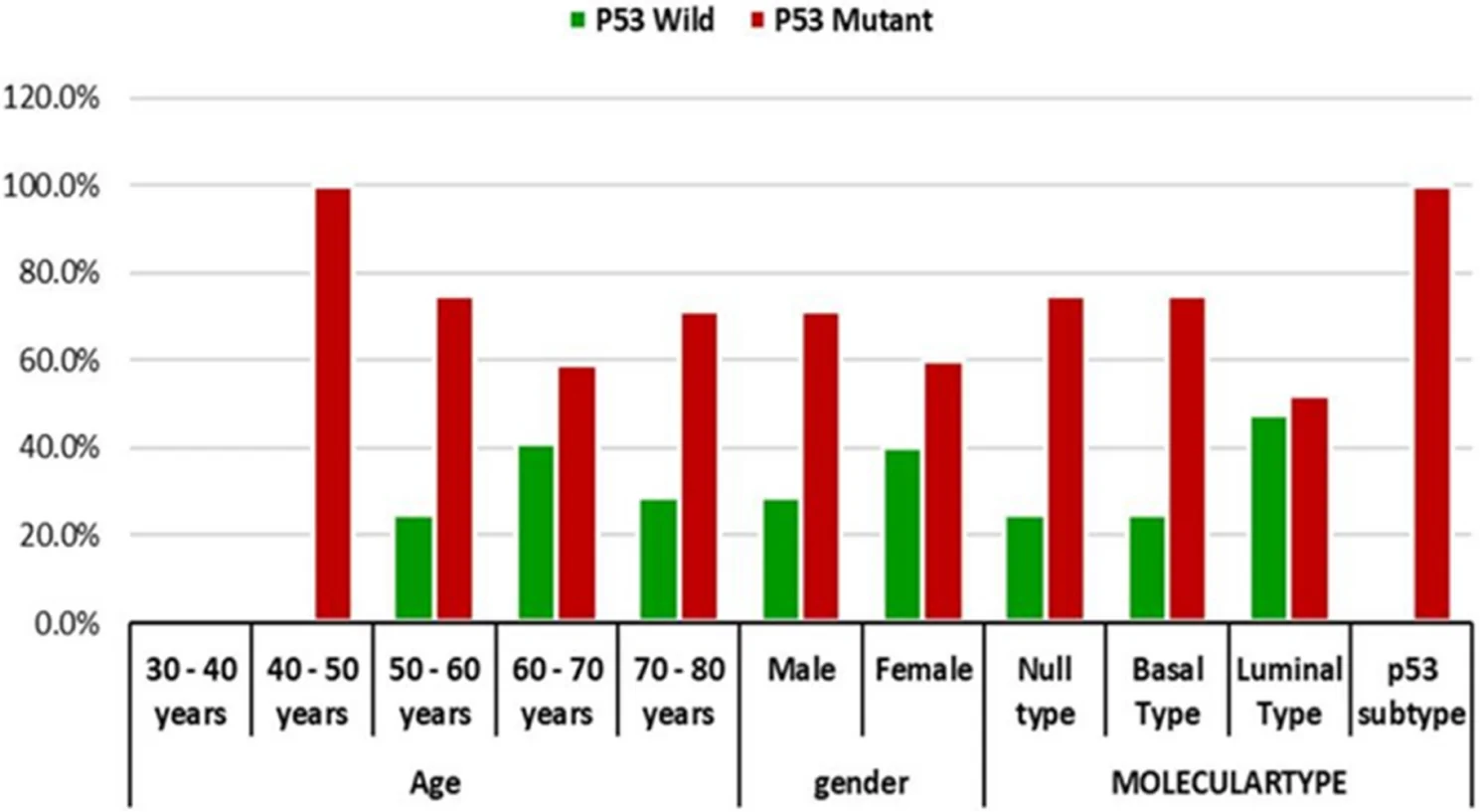
Showing p53 staining association with age, gender and molecular subtypes

No significant association was identified between p53 expression and age or gender (*p* = 0.408 and *p* = 0.597, respectively) (Table 4; Fig. 8). However, a statistically significant association between p53 expression and molecular subtype was observed (χ² = 8.80, *p* = 0.032). The p53-altered subtype demonstrated 100% aberrant p53 expression, whereas the luminal subtype showed an almost equal distribution of wild-type (47.8%) and mutant (52.2%) patterns. The basal and null subtypes demonstrated a higher proportion of mutant p53 cases (75%).

**Table 4:** Showing p53 staining association with age, gender and molecular subtypes

Variable		P53				Significance	
		Wild		Mutant			
		No.	%	No.	%	chisq	*p*-value
Age	30-40years	0	0.0%	0	0.0%	2.89	0.408
	40-50years	0	0.0%	4	100.0%		
	50-60years	3	25.0%	9	75.0%		
	60-70years	7	41.2%	10	58.8%		
	70-80years	4	28.6%	10	71.4%		
gender	Male	12	28.6%	30	71.4%	0.28	0.597
	Female	2	40.0%	3	60.0%		
MOLECULAR TYPE	Nulltype	1	25.0%	3	75.0%	8.80	**0.032**
	BasalType	2	25.0%	6	75.0%		
	LuminalType	11	47.8%	12	52.2%		
	p53subtype	0	0.0%	12	100.0%		

**Fig. 9:**
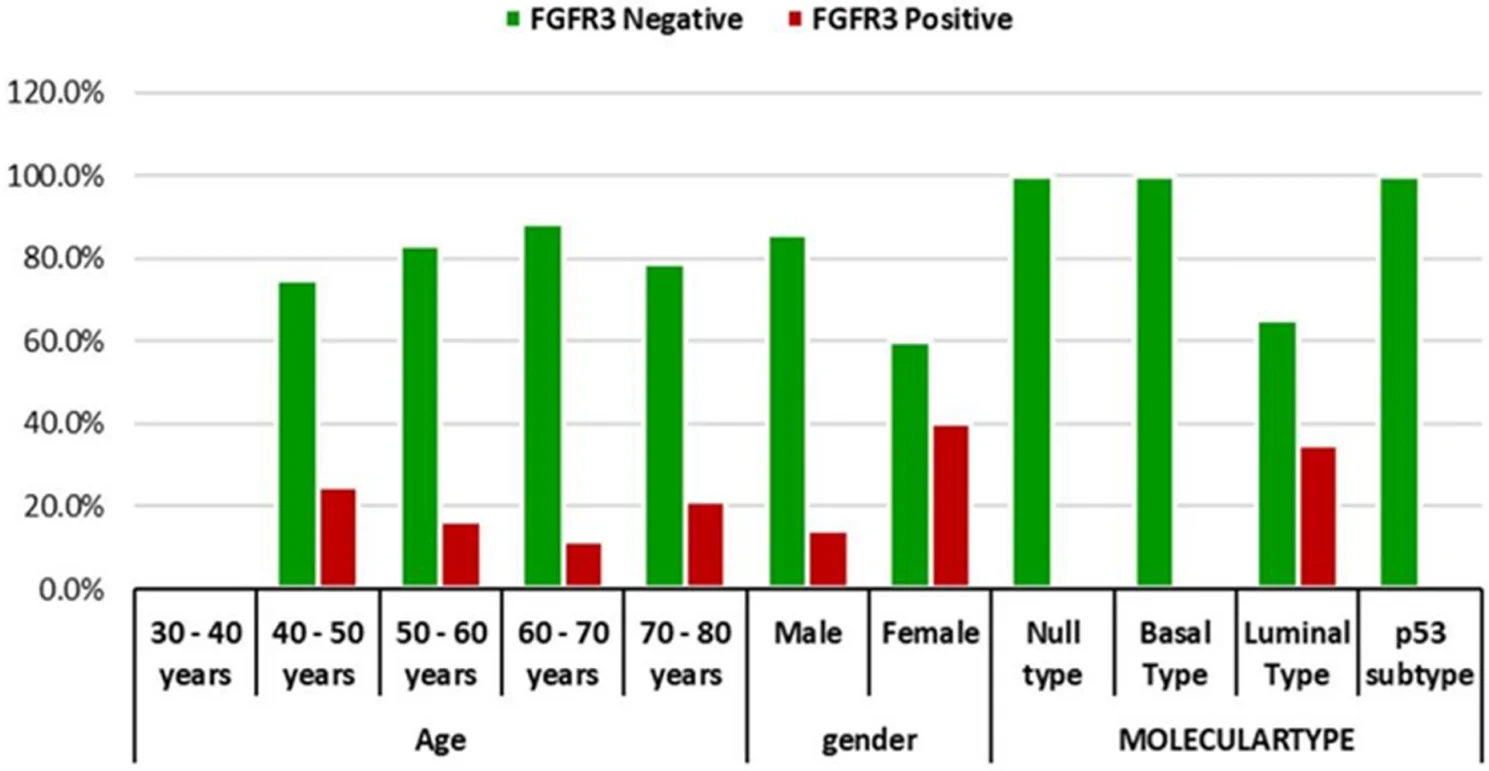
Association of FGFR3 with age, gender and molecular subtypes

FGFR3 expression was observed in 17% of cases and showed a significant association with molecular subtype (χ² = 10.06, *p* = 0.018) (Table 5; Fig. 9). Expression was predominantly restricted to the luminal subtype, where 34.8% of cases were FGFR3-positive.

**Table 5:** Association of FGFR3 with age, gender and molecular subtypes

Variable		FGFR3				Significance	
		Negative		Positive			
		No.	%	No.	%	chisq	*p*-value
Age	30-40years	0	0.0%	0	0.0%	0.71	0.872
	40-50years	3	75.0%	1	25.0%		
	50-60years	10	83.3%	2	16.7%		
	60-70years	15	88.2%	2	11.8%		
	70-80years	11	78.6%	3	21.4%		
gender	Male	36	85.7%	6	14.3%	2.09	0.148
	Female	3	60.0%	2	40.0%		
MOLECULAR TYPE	Nulltype	4	100.0%	0	0.0%	10.06	**0.018**
	BasalType	8	100.0%	0	0.0%		
	LuminalType	15	65.2%	8	34.8%		
	p53subtype	12	100.0%	0	0.0%		

In contrast, no FGFR3 positivity was observed in basal, p53-altered, or null subtypes, indicating a strong subtype-specific expression pattern.

No significant association was found between FGFR3 expression and age (*p* = 0.872) or gender (*p* = 0.148). FGFR3 positivity was highest in the 70–80 years age group (21.4%) and lowest in the 60–70 years group (11.8%). A higher rate of FGFR3 positivity was observed in females (40%) compared to males (14.3%).

**Fig. 10:**
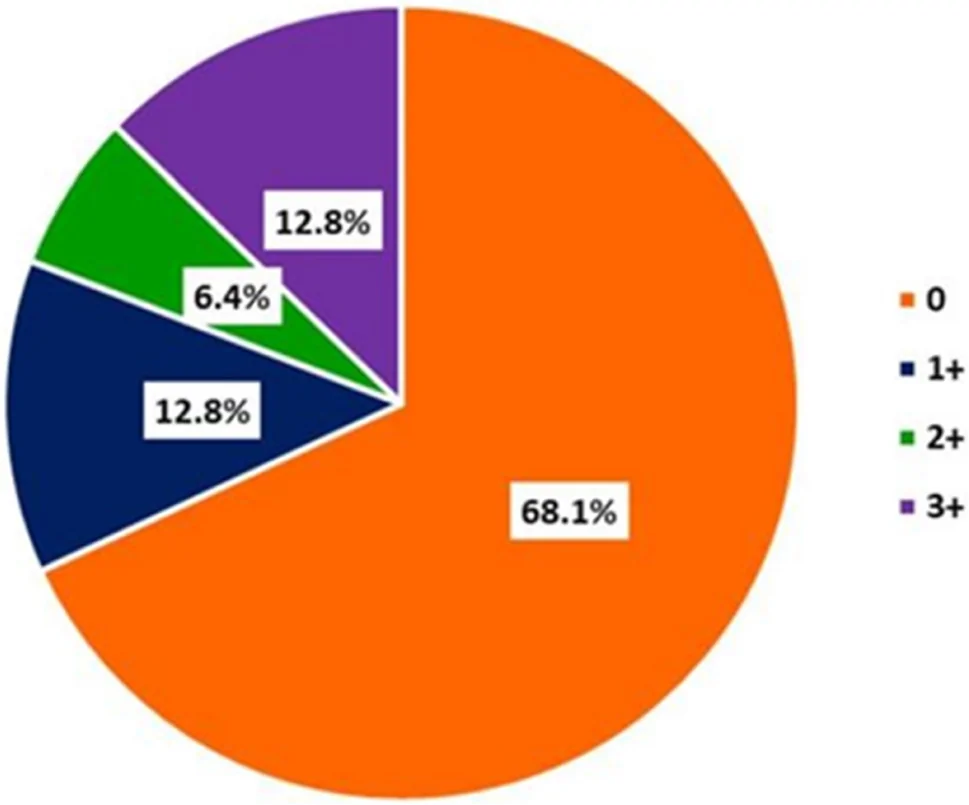
Distribution of HER2

HER2 immunohistochemistry revealed the following distribution(Table 6; Fig. 10)-.

- Score 0: 68.1%
- Score 1+: 12.8%
- Score 2+: 6.4%
- Score 3+: 12.8%

**Table 6:** Distribution of HER2 expression

HER2	No.	%
0	32	68.1%
1+	6	12.8%
2+	3	6.4%
3+	6	12.8%

**Fig. 11:**
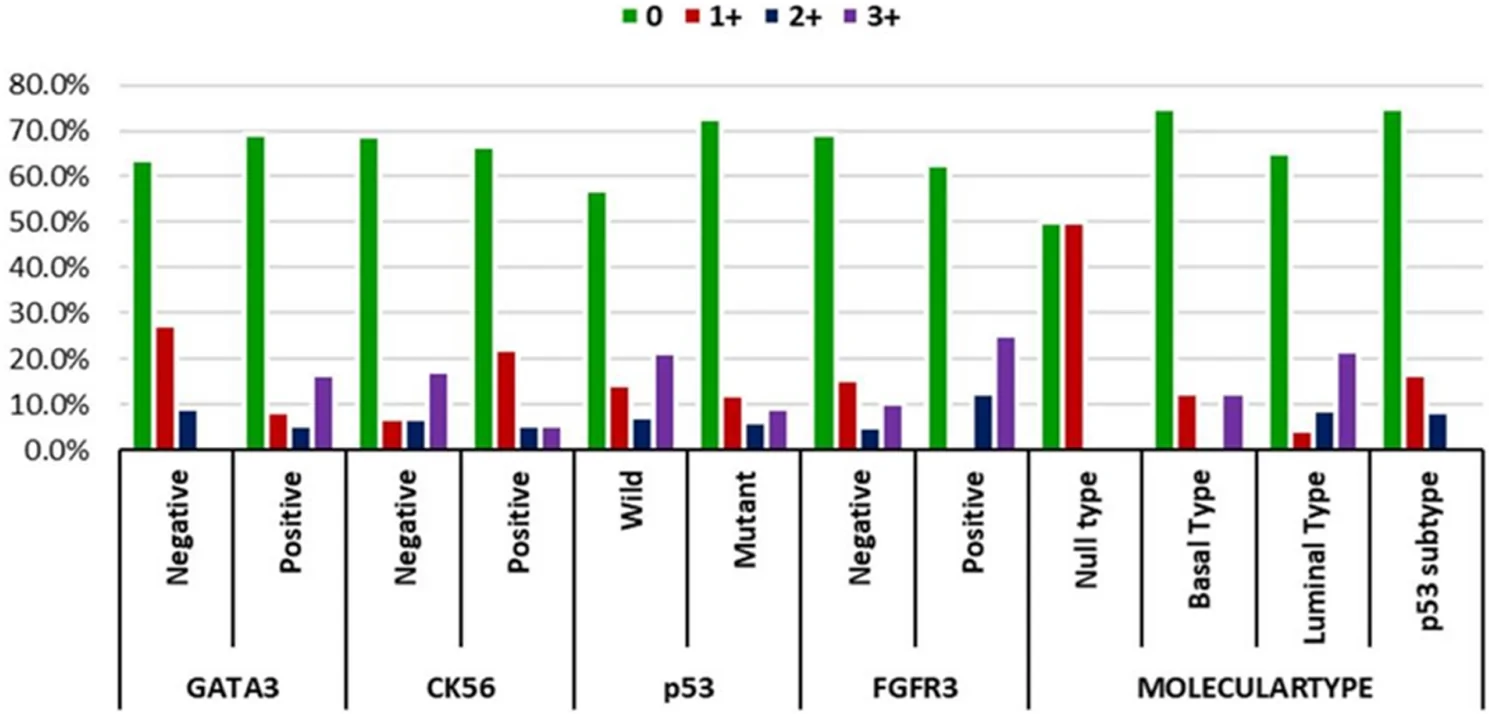
HER2 immunostaining association with other IHC and molecular types

Among molecular subtypes, HER2 overexpression (3+) was most frequently observed in the luminal subtype (21.7%), although this association did not reach statistical significance (χ² = 10.65, *p* = 0.301) (Table 7; Fig. 11).

**Table 7:** HER2 IHC association with other markers and molecular subtypes

Immunohistochemicalstains		HER2								Significance	
		0		1+		2+		3+			
		No.	%	No.	%	No.	%	No.	%	chi sq	*p*-value
GATA3	Negative	7	63.6%	3	27.3%	1	9.1%	0	0.0%	4.41	0.221
	Positive	25	69.4%	3	8.3%	2	5.6%	6	16.7%		
CK56	Negative	20	69.0%	2	6.9%	2	6.9%	5	17.2%	3.27	0.352
	Positive	12	66.7%	4	22.2%	1	5.6%	1	5.6%		
p53	Wild	8	57.1%	2	14.3%	1	7.1%	3	21.4%	1.58	0.665
	Mutant	24	72.7%	4	12.1%	2	6.1%	3	9.1%		
FGFR3	Negative	27	69.2%	6	15.4%	2	5.1%	4	10.3%	2.97	0.396
	Positive	5	62.5%	0	0.0%	1	12.5%	2	25.0%		
MOLECULAR TYPE	Null type	2	50.0%	2	50.0%	0	0.0%	0	0.0%	10.65	0.301
	Basal Type	6	75.0%	1	12.5%	0	0.0%	1	12.5%		

Correlation analysis between HER2 expression and individual biomarkers showed no statistically significant associations:

- GATA3 vs. HER2: χ² = 4.41, *p* = 0.221
- CK5/6 vs. HER2: χ² = 3.27, *p* = 0.352
- p53 vs. HER2: χ² = 1.58, *p* = 0.665
- FGFR3 vs. HER2: χ² = 2.97, *p* = 0.396

Despite the lack of statistical significance, a consistent trend of higher HER2 expression was observed in luminal tumors.

Overall, luminal tumors demonstrated frequent GATA3 positivity, along with higher expression of FGFR3 and HER2, supporting their differentiated phenotype. Basal tumors were characterized by CK5/6 expression, whereas p53-altered tumors exhibited widespread TP53 pathway abnormalities.

The null subtype lacked consistent expression of the evaluated markers, suggesting a biologically distinct group not fully captured by the current immunohistochemical panel.

## Discussion

Muscle-invasive bladder carcinoma (MIBC) represents a biologically and clinically heterogeneous malignancy in which tumor behaviour is driven by distinct molecular programs rather than purely histopathological features. Contemporary molecular studies have established that MIBC can be broadly categorized into luminal and basal subtypes, with additional subsets such as p53-like and double-negative tumors, reflecting divergent pathways of urothelial differentiation and oncogenic evolution [1, 5–8]. These molecular classes not only mirror normal urothelial biology but also exhibit distinct prognostic and therapeutic characteristics, thereby reshaping the conceptual framework of bladder cancer management.

In the present study, immunohistochemistry-based classification using a limited panel (GATA3, CK5/6, p53, and FGFR3) effectively stratified MIBC cases into molecular subtypes. This supports the growing consensus that immunohistochemistry can serve as a practical surrogate for transcriptomic profiling in routine diagnostic settings, particularly in resource-limited environments where high-throughput sequencing is not feasible [12, 13, 23–26]. The predominance of the luminal subtype observed in our cohort is consistent with several prior large-scale molecular classification studies, which have similarly reported luminal enrichment in MIBC populations [1, 5, 6, 10].

The biological distinction between luminal and basal tumors is well established. Luminal tumors typically express transcription factors such as GATA3 and exhibit urothelial differentiation programs analogous to luminal breast epithelium, whereas basal tumors are characterized by expression of high-molecular-weight cytokeratins (CK5/6) and a stem-like phenotype associated with increased proliferative capacity and invasiveness [1, 9, 27]. The strong correlation observed in our study between GATA3 expression and luminal subtype, and CK5/6 expression with basal subtype, reinforces the reliability of these markers in reflecting underlying tumor biology.

Importantly, molecular subtype classification carries significant prognostic and therapeutic implications. Basal tumors are frequently associated with higher-grade morphology, increased epithelial–mesenchymal transition (EMT) signatures, and more aggressive clinical behavior; however, they paradoxically demonstrate improved responsiveness to cisplatin-based neoadjuvant chemotherapy (NAC) [10, 11, 28, 29]. In contrast, luminal tumors often exhibit more differentiated histology but may display relative chemoresistance, necessitating alternative therapeutic approaches including targeted and immune-based strategies [10, 28, 29]. These observations underscore the clinical importance of molecular stratification in optimizing treatment selection.

### p53 pathway and molecular heterogeneity

Alterations in the p53 pathway represent a critical axis of urothelial carcinogenesis and contribute significantly to tumor heterogeneity in MIBC. In the present study, p53 aberrations were frequently identified and demonstrated a significant association with molecular subtype, particularly within the luminal group. Although no significant correlation was observed with age or gender, the distribution pattern suggests biological rather than demographic determinants of p53 dysregulation.

The concept of a “p53-like” subtype, originally described by the MD Anderson classification, identifies tumors with intact histological features but dysfunctional p53 signaling, often associated with resistance to chemotherapy [9, 15, 16]. These tumors exhibit a paradoxical phenotype in which luminal differentiation is retained, yet genomic instability and chemoresistance pathways are activated. Our findings are consistent with previous studies demonstrating enrichment of p53 alterations within luminal tumors and support the hypothesis that p53 dysfunction contributes to therapeutic resistance and disease progression in this subgroup [15–18].

Furthermore, large-scale genomic analyses, including The Cancer Genome Atlas (TCGA), have confirmed that p53 pathway alterations are among the most frequent molecular events in MIBC and are strongly associated with aggressive disease biology [16, 17]. Collectively, these findings reinforce the role of p53 not merely as a prognostic marker but as a functional determinant of treatment response.

### FGFR3 expression and therapeutic implications

FGFR3 expression in the present study was significantly enriched in the luminal subtype, consistent with established molecular classifications of urothelial carcinoma. FGFR3 alterations are classically associated with low-grade non–muscle-invasive tumors; however, emerging evidence indicates that FGFR3 expression also persists in a subset of MIBC, particularly within luminal tumors [14, 28, 29].

This observation has important translational implications, as FGFR3 has emerged as a clinically actionable target in urothelial carcinoma. The development of FGFR inhibitors has introduced new therapeutic opportunities for patients harboring FGFR-altered tumors [14, 15, 28, 29]. Our findings therefore support the integration of FGFR3 testing into routine molecular profiling, particularly in luminal MIBC, where targeted therapy may complement or replace conventional chemotherapy in selected cases.

Although the overall frequency of FGFR3 positivity in this study was modest, its strong subtype specificity suggests a biologically distinct luminal subset with potentially targetable oncogenic dependency. This aligns with previous studies reporting variable FGFR3 prevalence across cohorts but consistent enrichment in luminal tumors [30–32].

### HER2 expression and emerging targeted therapy relevance

HER2 expression analysis in this study demonstrated predominant localization within the luminal subtype, with a smaller proportion of cases showing strong (3+) expression. This finding is consistent with increasing evidence that HER2 alterations are enriched in luminal urothelial carcinomas and may represent a distinct molecular subset with therapeutic relevance [18–20, 33].

Although HER2-targeted therapies are well established in breast and gastric cancers, their role in urothelial carcinoma remains investigational but promising [21, 22, 31]. Early clinical and translational studies suggest that HER2 overexpression or amplification may identify patients who could benefit from targeted agents, antibody–drug conjugates, or combination strategies [21, 22]. The preferential enrichment of HER2 positivity in luminal tumors observed in our cohort further supports the hypothesis that luminal MIBC constitutes a therapeutically actionable subtype.

Previous studies, including those by Kiss et al. and Campobasso et al., have similarly demonstrated higher HER2 expression in luminal tumors compared to basal tumors, reinforcing the reproducibility of this association across independent cohorts [4, 31]. These findings collectively highlight the potential role of HER2 as both a prognostic and predictive biomarker in MIBC.

### Null/double-negative subtype and biological complexity

A subset of tumors in our study exhibited a null or double-negative phenotype, lacking expression of both luminal and basal markers. This subgroup, although relatively small, represents an important biological category that may reflect either technical limitations of IHC-based classification or a distinct molecular entity with alternative differentiation pathways. Similar null-type tumors have been reported in previous studies, including those by López-Beltrán et al., who identified double-negative phenotypes in a minority of MIBC cases [34]. The existence of this subgroup underscores the inherent complexity of bladder cancer biology and suggests that a proportion of tumors may not conform to conventional luminal–basal dichotomy [35, 36]. Such cases may require comprehensive genomic profiling to fully elucidate their molecular drivers and therapeutic vulnerabilities.

### Clinical implications and study significance

Collectively, the findings of this study reinforce the clinical utility of immunohistochemistry-based molecular subtyping in MIBC. The ability to stratify tumors into biologically meaningful categories using a limited marker panel provides a cost-effective and clinically accessible approach for routine pathology practice [12, 13, 23–25]. More importantly, the observed associations between molecular subtypes and biomarkers such as p53, FGFR3, and HER2 highlight the potential for integrated molecular–immunohistochemical profiling to inform personalized therapeutic strategies.

Such an approach is particularly relevant in the era of precision oncology, where treatment decisions are increasingly guided by tumor biology rather than histological classification alone. Integration of molecular subtyping into clinical workflows may facilitate improved prediction of chemotherapy response, identification of candidates for targeted therapy, and ultimately optimization of patient outcomes [6, 21, 22].

Immune checkpoint inhibitors have shown long term durable response and tolerable safety profiles in several clinical trials. Therefore, further research on combining immune checkpoint inhibitor therapy with other therapeutic modalities, such as cytotoxic chemotherapy or different therapeutic targets, is needed to reinforce the effects of immunotherapies [37]. The success of immune checkpoint therapy in advanced UC, as well as the precedent of neoadjuvant approach toward conventional chemotherapy, has led to significant interest in exploring the potential role of neoadjuvant Immune checkpoint blockade (ICB) in MIBC. Neoadjuvant immune checkpoint blockade did not delay planned surgery. Checkpoint inhibitor monotherapy was well tolerated, with no unexpected toxicity in the presurgical setting showing complete response(CR) ranging from 31% with an anti–PD-L1 monoclonal antibody (mAb) atezolizumab (*n* = 27/88) to 37% with anti–PD-1 mAb pembrolizumab (*n* = 42/ 114). Overall, 92% (*n* = 87/95) and 98% (*n* = 112/114) of the patients underwent radical cystectomy Early phase I/II trials investigating neoadjuvant combination chemotherapy strategies with immune checkpoint blockers reported enhanced anti tumor efficacy, with a pathological CR ranging from 33% to 50% [38–40]. 

### Limitations

The principal limitation of this study is its relatively small sample size, which may limit statistical power and generalizability. Additionally, the absence of long-term survival data restricts the ability to correlate molecular subtypes with clinical outcomes. Future multicentre studies with larger cohorts and integrated genomic analysis are warranted to validate these findings and refine the clinical utility of immunohistochemical subtyping.

Prospective studies evaluating treatment response stratified by molecular subtype, particularly in the context of neoadjuvant chemotherapy and emerging targeted therapies, will be critical in translating molecular classification into clinical decision-making tools.

## Conclusion

This study provides valuable insights into the molecular classification of Muscle Invasive Bladder Carcinoma (MIBC) using immunohistochemistry and explores its association with HER2 expression. The most commonly affected age group was 60–70 years, with a higher incidence observed in males. Among the molecular subtypes, the luminal type was the most prevalent. GATA3 expression was predominantly associated with the luminal subtype, while CK5/6 positivity characterized the basal subtype.

Although p53 expression did not show a significant correlation with age or gender, a meaningful association was observed with molecular subtypes, particularly in the luminal group. Similarly, FGFR3 expression was more frequent in the luminal subtype, without any notable age or gender correlation. HER2 expression was also most commonly seen in the luminal group. Therefore, adapting HER2-targeting therapy in the context of molecular subtypes could be a promising strategy mainly in luminal subtype.

The limited sample size was a constraint of this study, restricting the generalizability of the findings. Larger studies involving more diverse patient populations and extended follow-up periods are essential to validate these results. Further research focusing on long-term outcomes and predictive value of marker expression patterns could enhance the development of targeted therapies, ultimately improving disease-free and overall survival in patients with MIBC.

## Data Availability

All the Data were obtained from the requisition form, case records and hospital’s Laboratory information system (LIS). All data generated or analyzed during this study are included in this published article.

## Abbreviations

UC: Urothelial carcinoma
NMIBC: Non-muscle invasive bladder cancer
MIBC: Muscle-invasive bladder cancer
TURBT: Transurethral resection of the bladder tumor
